# Frequency-dependent effects of subthalamic deep brain stimulation on motor symptoms in Parkinson’s disease: a meta-analysis of controlled trials

**DOI:** 10.1038/s41598-018-32161-3

**Published:** 2018-09-27

**Authors:** Dongning Su, Huimin Chen, Wanli Hu, Yuye Liu, Zhan Wang, Xuemei Wang, Genliang Liu, Huizi Ma, Junhong Zhou, Tao Feng

**Affiliations:** 1 Department of Movement Disorders, Center for Neurology, Beijing Tiantan Hospital, Capital Medical University, Beijing, China; 2 China National Clinical Research Center for Neurological Diseases, Beijing, China; 3 Department of Hematology and Oncology, Jingxi Campus, Beijing ChaoYang Hospital, Capital Medical University, Beijing, China; 4 Department of Functional Neurosurgery, Beijing Neurosurgical Institute, Capital Medical University, Beijing, China; 5 Hebrew Seniorlife Institution for Aging Research, Harvard Medical School, Boston, Massachusetts USA

## Abstract

This study aims to investigate how the frequency settings of deep brain stimulation (DBS) targeting the subthalamic nucleus (STN) influence the motor symptoms of Parkinson’s disease (PD). Stimulation with frequencies less than 100 Hz (mostly 60 or 80 Hz) is considered low-frequency stimulation (LFS) and with frequencies greater than 100 Hz (mostly 130 or 150 Hz) is considered high-frequency stimulation (HFS). We conducted a comprehensive literature review and meta-analysis with a random-effect model. Ten studies with 132 patients were included in our analysis. The pooled results showed no significant difference in the total Unified Parkinson Disease Rating Scale part III (UPDRS-III) scores (mean effect, −1.50; p = 0.19) or the rigidity subscore between HFS and LFS. Compared to LFS, HFS induced greater reduction in the tremor subscore within the medication-off condition (mean effect, 1.01; p = 0.002), while no significance was shown within the medication-on condition (mean effect, 0.01; p = 0.92). LFS induced greater reduction in akinesia subscore (mean effect, −1.68, p = 0.003), the time to complete the stand-walk-sit (SWS) test (mean effect, −4.84; p < 0.00001), and the number of freezing of gait (FOG) (mean effect, −1.71; p = 0.03). These results suggest that two types of frequency settings may have different effects, that is, HFS induces better responses for tremor and LFS induces greater response for akinesia, gait, and FOG, respectively, which are worthwhile to be confirmed in future study, and will ultimately inform the clinical practice in the management of PD using STN-DBS.

## Introduction

Parkinson’s disease (PD) is a neurodegenerative disorder, characterized by pathological motor symptoms including tremors, rigidity, bradykinesia and postural instability^1^. After an initial honeymoon period, pharmacological treatments often fail to alleviate the burden from those symptoms, severely compromising the quality of life for PD patients. The pathological symptoms in PD may arise at least in part due to the dysfunction in thalamic region in the brain. Deep brain stimulation (DBS) targeting the subthalamic nucleus (STN) has been evidenced as an effective intervention to improve the functional performance in those suffering from advanced PD. The STN-DBS modulates the activity of certain nucleus via implanted electrodes and thus disrupts the pathologic oscillations of alpha- (8–12 Hz), beta- (12–30 Hz), and gamma-(30–100 Hz) bands within the cortico-basal ganglia loop^2–5^. Previous studies showed that STN-DBS can improve the functional performance of PD patients as evidenced by a 25% decrease in the Unified Parkinson Disease Rating Scale part III (UPDRS-III) scores, a 25% decrease in the average levodopa-equivalent daily dose (LEDD) and an 80% decrease in drug-induced dyskinesia^6^.

The effectiveness of STN-DBS on PD depends mainly upon the parameters used in the intervention, including the intensity, frequency, pulse width (PW) and contact configuration^7^. No guidelines are currently available to determine the optimal parameters of the DBS intervention. Only the impact of the intensity (i.e., amplitude of the applied current) and pulse width on the intervention effects are fairly well-understood. For example, the high voltage and/or narrow PW (e.g., 30 µs) of stimulation can induce greater effects (e.g., longer therapeutic window) compared to low voltage and/or wide PW^8,9^. However, the influence of the frequency settings on the therapeutic effects of STN-DBS on motor symptoms are still not fully understood.

The frequency of DBS is often categorized as high frequency (i.e., HFS, >100 Hz, mostly 130 or 150 Hz) or low frequency (i.e., LFS, <100 Hz, mostly 60 or 80 Hz)^1^. These two categories have varied therapeutic effects on motor function in those with PD. For example, Khoo *et al*. reported that LFS induced greater improvements in motor control performance (i.e., lower UPDRS scores, particularly in akinesia and axial subscores) compared to HFS. Xie *et al*. showed using LFS significantly improved aspiration, swallowing, FOG and axial symptoms^10,11^. However, Vallabhajosula *et al*. observed no significant improvements in total UPDRS-III scores, step length and velocity during gait initiation, and gait speed after LFS^12^. As such, no consensus on the effects of STN-DBS frequency settings has been reached, and the potential roles of frequency in ameliorating motor symptoms in PD may be underestimated.

Therefore, we here completed a meta-analysis to quantitatively analyze the acute effects (i.e., several minutes to hours) of LFS and HFS of STN-DBS on motor symptoms in PD patients based upon previously published studies. This work may provide us a better understanding on the influences of the frequency settings on motor symptoms in PD, which will ultimately optimize the frequency-programming protocols of STN-DBS for different motor symptoms in clinical practice.

## Methods

### Search criteria

We conducted a comprehensive review of the published literature reporting the acute effects of LFS and HFS in STN-DBS on motor symptoms in PD. The Pubmed, Embase, the Cochrane Library and the Web of Science were used for the literature research. We reviewed publications up to February 2018 by searching citing and cited articles. We also checked cross-references for certain crucial articles. We used a combination of MeSH and text word searching for the following terms: “(deep brain stimulation OR electrical stimulation OR neuromodulation or DBS) AND frequency AND (subthalamic nucleus OR STN) AND (Parkinson’s disease OR PD)”.

### Eligibility criteria

The inclusion criteria for the searched studies were: written in English; focusing on human participants; participants with PD; unilateral or bilateral procedures; STN-DBS; prospective studies; comparison between LFS (<100 Hz, mostly 60 or 80 Hz) and HFS (>100 Hz, mostly 130 or 150 Hz); UPDRS-III, stand-walk-sit (SWS) test and FOG questionnaire as measurements. The exclusion criteria were: DBS procedure as a treatment for diseases other than PD; targeting other than STN or combined targets; less than 5 participants; retrospective analyses; case reports; review articles; editorials; letters; conference articles. The Prospero registration number of this study is 42017060545.

### Quality evaluation and data collection

Quality evaluation was conducted according to the Cochrane Collaboration’s tool for assessing the risk of bias by two physicians separately. Subsequently, study details were extracted from the retrieved studies, including the number of patients, mean age, duration of disease, study designs, post-surgery duration, medication status (on and off), time for adapting to the changed stimulation conditions. The means and standard deviations (SDs) of UPDRS-III scores and subscores, as well as the time to complete SWS tests were collected. The means and SDs were calculated for those data presented as medians and range values as previously described^13^. These procedures were also conducted by two physicians independently to ensure accuracy of analyses.

### Statistical analysis

The acute effects of LFS and HFS on motor performance, tremors, rigidity, gait and akinesia were assessed by the changes in the corresponding subscores in the UPDRS III. The results of the SWS test were also summarized. Using Review Manager Version 5.3, a random-effect meta-analysis of continuous variables was performed to pool the mean effect and 95% confidence intervals (CI) for a more conservative estimate of pooled effects. The Cochran’s Q-test and I2-values were adapted for assessment of statistical heterogeneity between studies. Heterogeneity was regarded as mild, moderate and high separately when the I2 was above 25%, 50% and 75%, respectively^14^. The extent of DBS-induced improvement in motor symptoms may possibly influenced by the medication states (i.e., on and off)^15,16^. But due to the limited number of studies within the medication-on state, we only summarized and analyzed the data within the medication-off condition. No publication bias analysis was performed due to the low number of included studies.

## Results

### Study identification and characteristics of included studies

**Figure 1:**
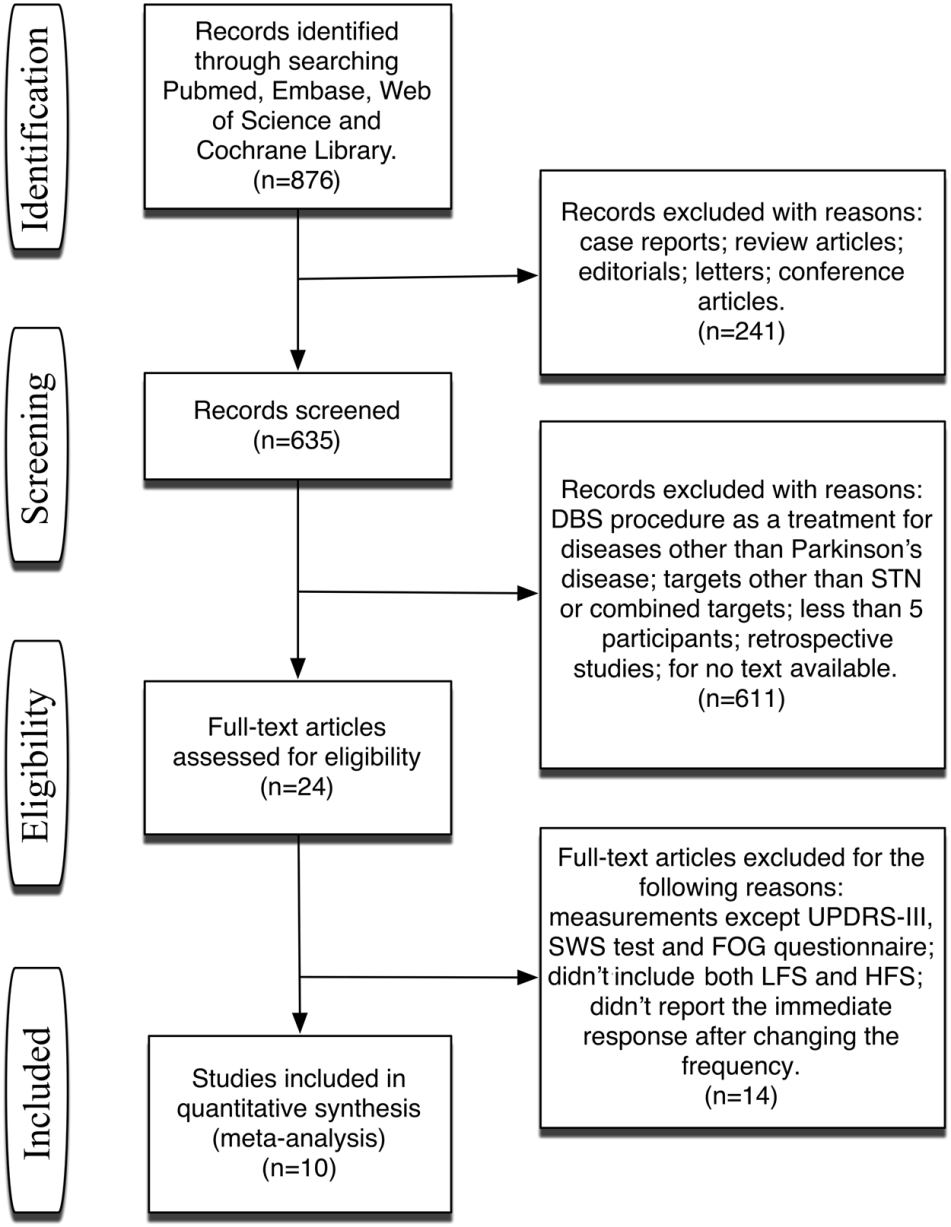
Flow chart of literature search and study selection.

**Figure 2:**
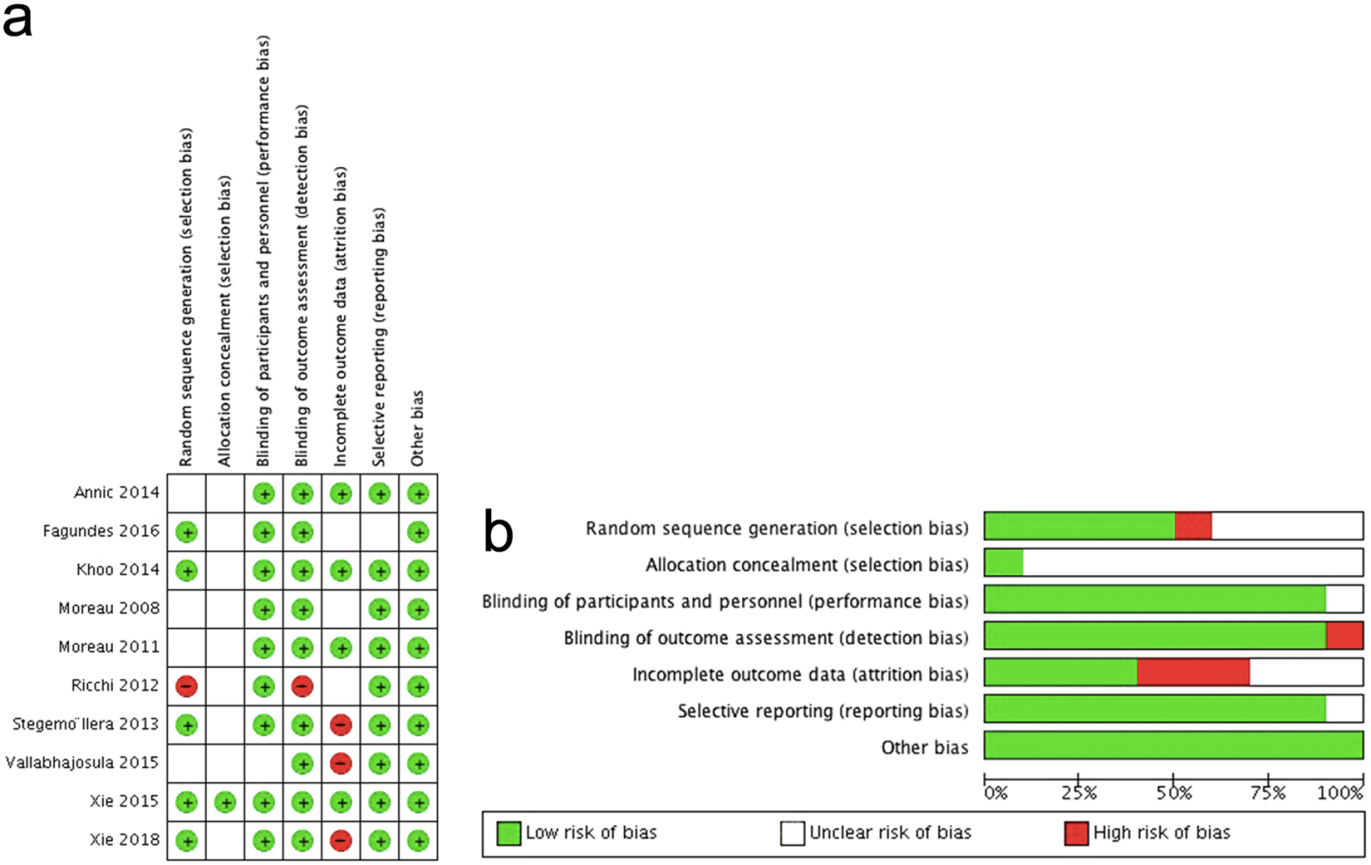
Risk-of-bias assessment of included studies.

We identified a total of 876 records from databases following our search strategy. After reviewing titles and abstracts, we excluded 241 unqualified records. We then retrieved full-text articles and found 24 studies that compared the acute effects of low- and high- frequency STN-DBS on motor symptoms in PD. Ten of them met the inclusion criteria (Fig. 1). We then evaluated the allocation, blinding, incomplete outcome data, selective reporting, and other potential sources of bias of the 10 eligible studies (Fig. 2).

Four out of the ten studies were completed under the medication-on condition^10,11,17^, five were completed under the medication-off condition^12,18–20^, and the other one did not separate different medication conditions. The age of participants ranged from 31 to 76 years old, and their history of PD ranged from 8 to 29 years. Time intervals between DBS parameter adjustment and motor performance evaluation ranged from 10 to 180 minutes. Among these studies, UPDRS-III scores were reported in 9 studies, and the results of SWS tests were reported in 4 studies. Characteristics of the included studies are presented in Table 1.

**Table 1:** Characteristic of studies included in the meta-analysis.

Author & Year	Partici-pants	Mono/Bi	Drugs	Age	Disease duration (years)	Post-surgery time (months)	Measurements	Intervals between adjustment of parameters and evaluation (minutes)	Design of changing frequency
Xie *et al*.^26^	11	bilateral	on	68.50 ± 5.90	14.2 ± 5.7	42 ± 48	UPDRS-III, VFSS studies, the Penetration-Aspiration Scale, FOG questionnaire score and SWS test.	30	usual voltage and pulse-width
Fagundes *et al*., 2016	20	bilateral	off/on	56.65 ± 10.71	15.3 ± 4.71	2.21 ± 1.38	VF tasks, URDPS-III.	60	usual voltage and pulse-width
Vallabhajosula *et al*.^12^	19	bilateral	washed out	61.80 ± 9.00	13.60 ± 4.20	39.21 ± 23.67	UPDRS-III, the Vicon’s Plug-in-Gait marker system.	10	usual voltage and pulse-width
Xie *et al*.^11^	7	bilateral	on	64.00 ± 8.00	12.90 ± 4.90	52.8 ± 58.8	MBS studies, the Penetration-Aspiration Scale ratings, UPDRS-III, FOG questionnaire score and SWS test.	30	usual voltage and pulse-width
Khoo *et al*.^10^	14	bilateral	on	60.86 ± 9.28	16.00 ± 4.99	24.36 ± 17.30	UPDRS- III, UPDRS-III subscores, 10-meter timed walk test, BBS.	60	constant TEED
Annic *et al*.^18^	22	bilateral	washed out	—	17.00 (Q1–Q3: 15.00–22.00)	68.40 (Q1–Q3: 48.00–81.60)	SWS test, UPDRS-III, GABS score.	60	constant TEED together with constant pulse width
Stegemo llera *et al*.^19^	17	bilateral	washed out	61.53 ± 9.22	13.59 ± 3.91	30.53 ± 19.26	UPDRS-III; gait testing; balance; verbal fluency.	10	usual voltage and pulse-width
Ricchi *et al*.^17^	11	—	on	62.90 ± 4.30	—	54.00 ± 16.80	SWS test, UPDRS-II and -III.	180	constant TEED
Moreau *et al*., 2011	11	bilateral	washed out	69.00	19.00 (17.00–23.00)	60.00 (36.00–96.00)	UPDRS item 18,the IS, Voice recordings.	60	constant TEED with constant pulse width
Moreau *et al*.^20^	13	bilateral	washed out	70.00 (Q1–Q3: 66.00–72.00)	18.00 (Q1–Q3: 13.00–22.00)	60.00 (Q1–Q3: 48.00–60.00)	SWS test, UPDRS-III.	60	constant TEED with constant pulse width

The results are presented as mean ± SD/ median/median (minimum to maximum)/median (Q1–Q3). Abbrevations: Q1, first quartile; Q3, third quartile; VF, verbal fluency; VFSS, video fluoroscopic swallow; UPDRS, Unified Parkinson’s Disease Rating Scale; MBS, modified barium swallow; FOG, freezing of gait; SWS, stand-walk-sit; BBS, berg balance scale; IS, speech intelligibility score; TEED, total electrical energy delivered; GABS, gait and balance scale.

### Overall motor performance

**Figure 3:**
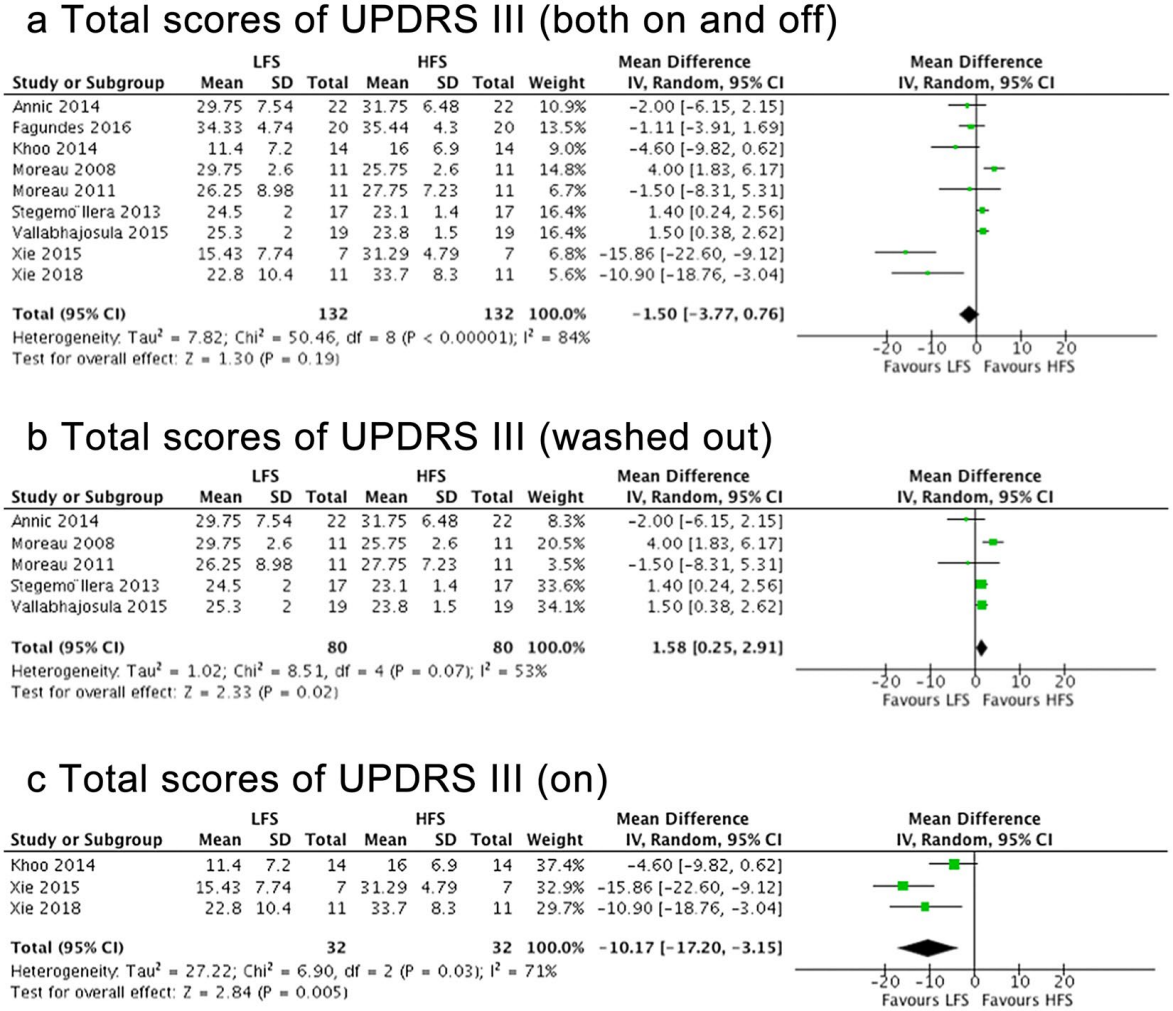
Forest plot of comparisons. Total scores of UPDRS-III with different medication conditions.

No significant difference was found between LFS and HFS on total UPDRS-III scores across all studies (Fig. 3a). Within the medication-off state, UPDRS-III scores improved more after using HFS DBS compared to LFS (mean effect, 1.58; 95% CI, 0.25–2.91; p = 0.02) (Fig. 3b). However, under the medication-on condition, LFS was more effective than HFS (mean effect, −10.17; 95% CI, −17.2–3.15; p = 0.005) (Fig. 3c). The inconsistency among studies was moderate to high (Q = 8.51, I^2^ = 53% and Q = 6.9, I^2^ = 71%).

### Tremor

**Figure 4:**
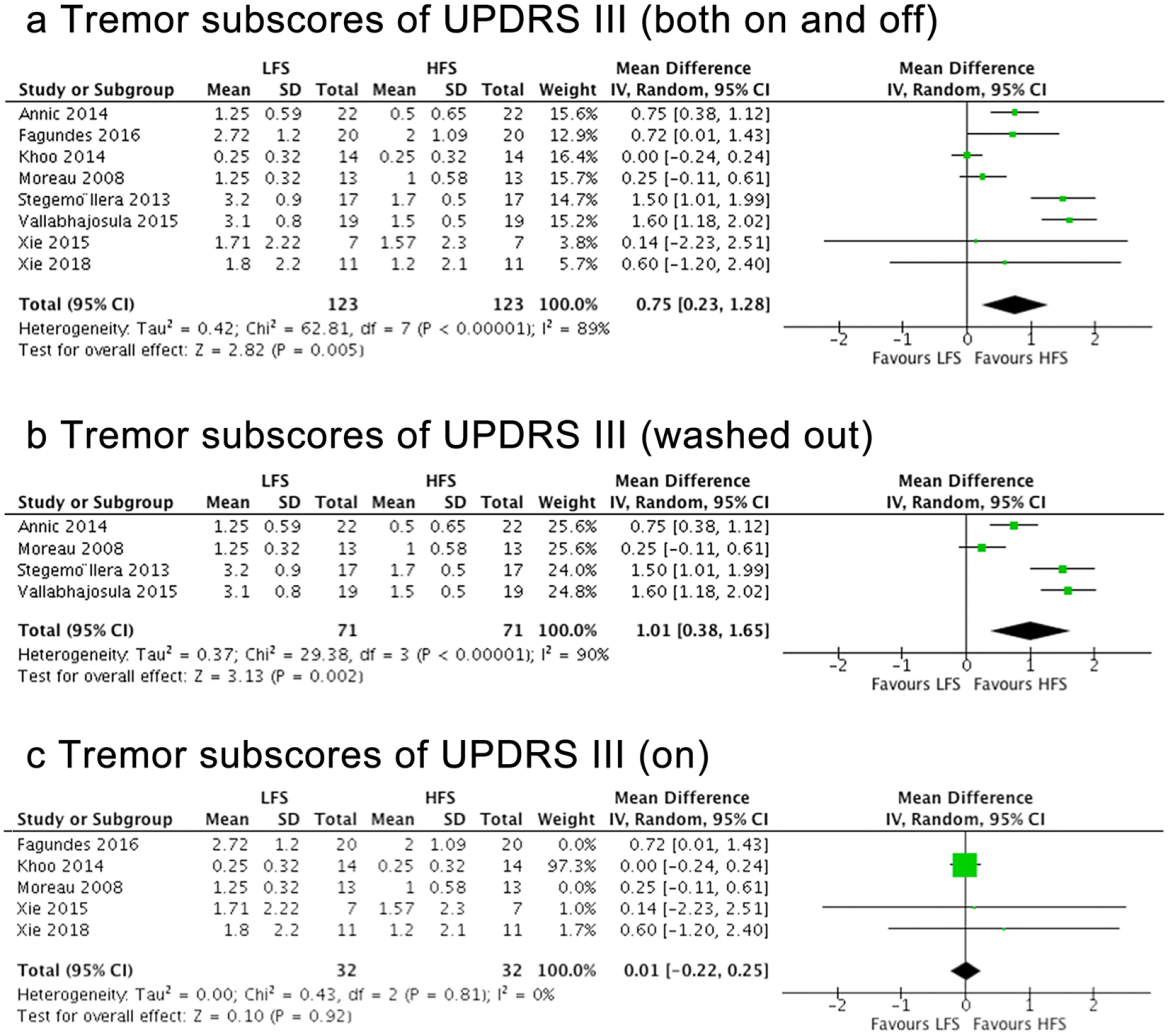
Forest plot of comparisons. Tremor subscores of UPDRS-III with different medication conditions.

Eight studies, consisting of 123 patients, used Item 20 and/or Item 21 in UPDRS-III to measure tremors. HFS had better effects on tremors than LFS across all eight studies when combining studies in both medication-on and -off states (mean effect, 0.75; 95% CI, 0.23–1.28; p = 0.005) (Fig. 4a). Particularly, within the medication-on condition (four studies included), significant effects of HFS on tremor were observed compared to LFS (mean effect, 1.01; 95% CI, 0.38–1.65; p = 0.002) (Fig. 4b), while, within the medication-off condition, no significant differences were found between LFS and HFS (mean effect, 0.01; 95% CI, −0.22–0.25; p = 0.92) (Fig. 4c). The inconsistency among the studies was low for the studies in medication-on condition (Q = 0.43, I^2^ = 0%) and it was relatively high for those in medication-off condition (Q = 29.38, I^2^ = 90%).

### Gait

Based upon the gait subscores of UPDRS-III in the medication-off condition and the time to complete the SWS test in the medication-on condition, LFS was more effective on gait than HFS (mean effect, −0.07; 95% CI, −0.13 to 0; p = 0.04 and mean effect, −4.84; 95% CI, −6.89- to −2.80; p < 0.00001) (Fig. 6b,d). The inconsistency was low across these studies (Q = 2.22, I^2^ = 10% and Q = 0.08, I^2^ = 0%).

**Figure 5:**
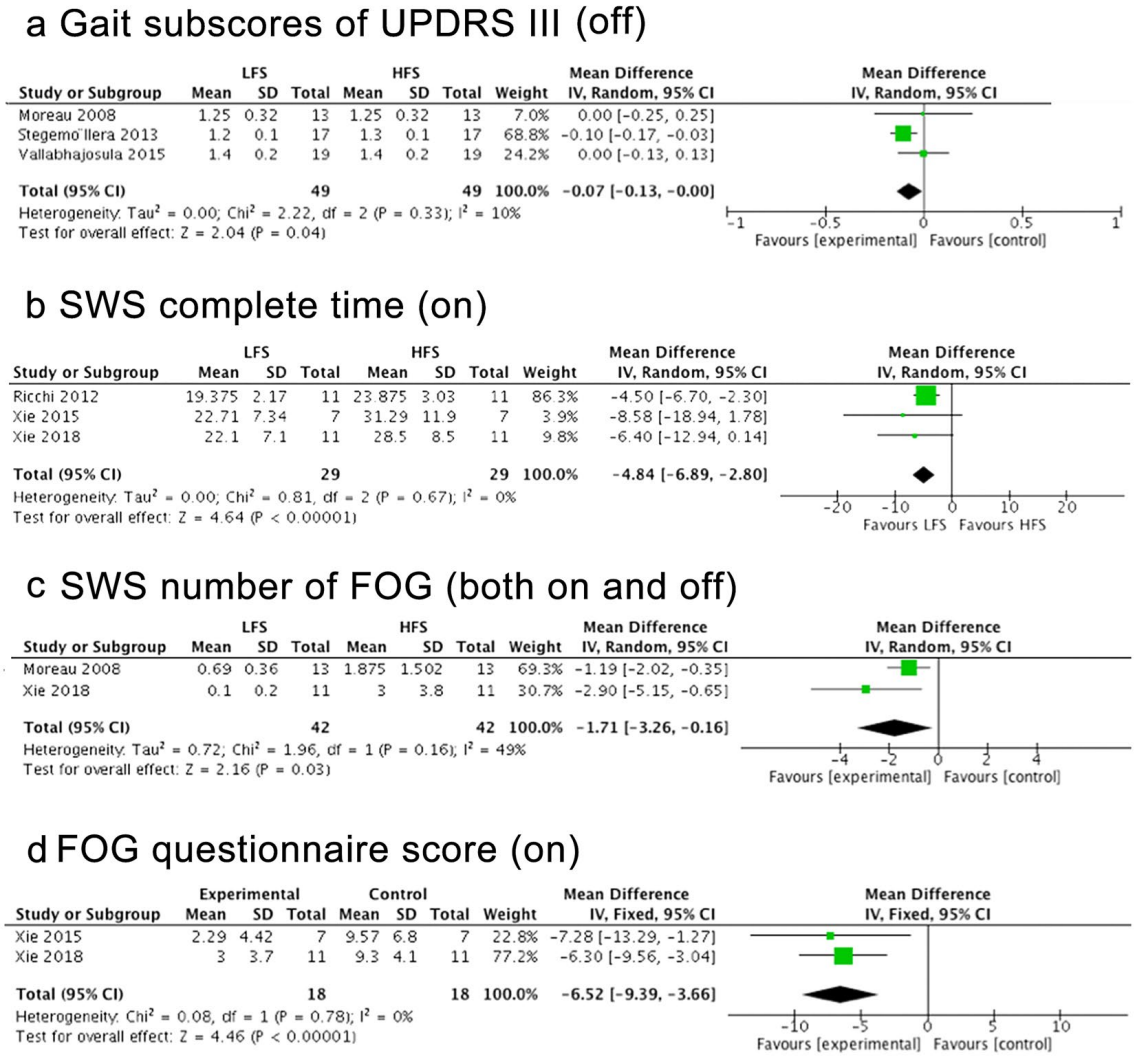
Forest plot of comparisons. Gait subscores of UPDRS-III, time to complete SWS tests, FOG numbers and scores of FOG questionnaire with different medication conditions.

**Figure 6:**
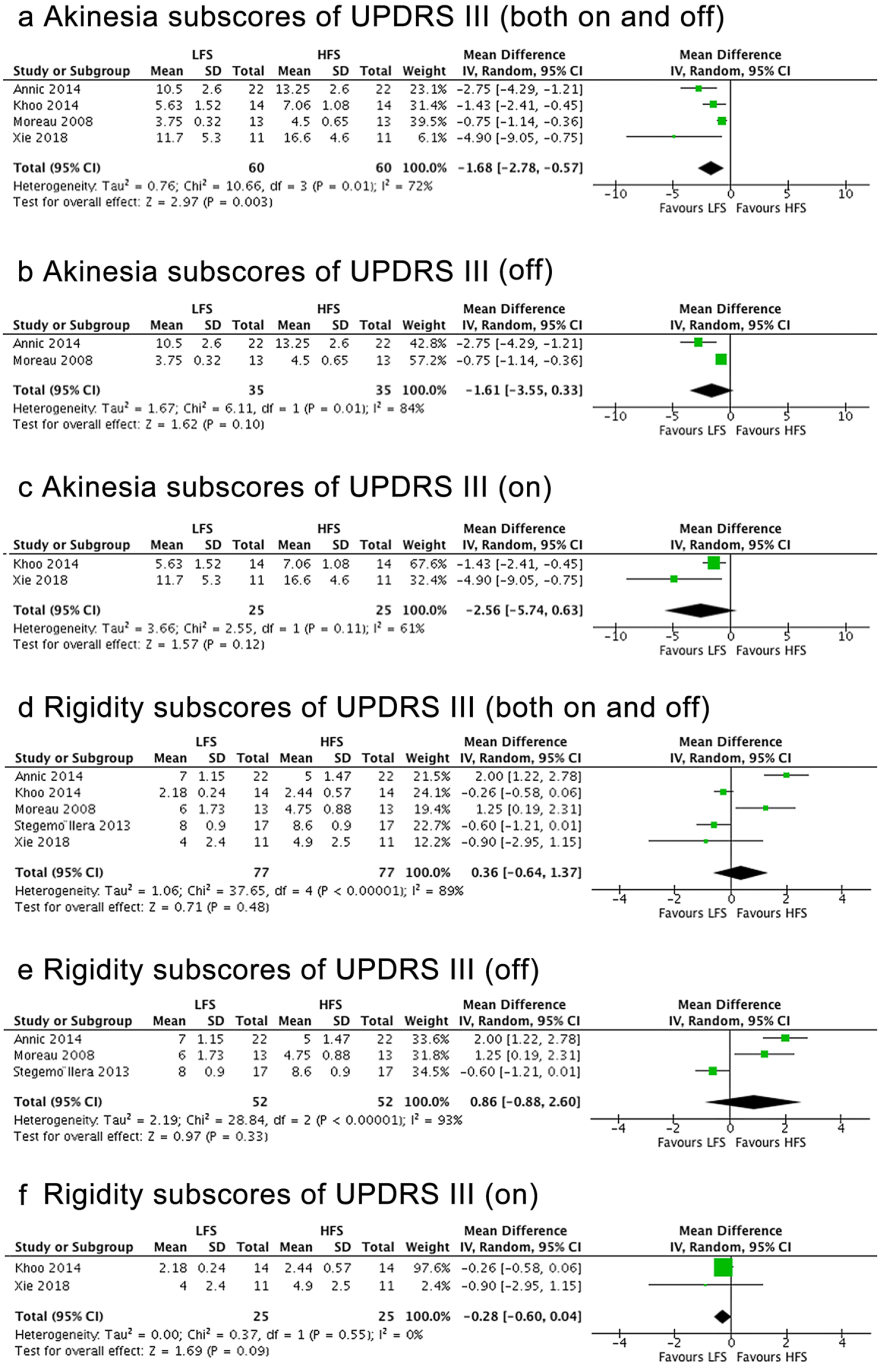
Forest plot of comparisons. Akinesia and rigidity subscores of UPDRS-III with different medication conditions.

### Freezing of gait

LFS induced greater reduction in FOG of PD compared to HFS. Specifically, the number of FOG during SWS tests (mean effect, −1.71; 95% CI, −3.26 to −0.16; p = 0.03, Fig. 6e) and the scores of FOG questionnaires (mean effect, −6.52; 95% CI, −9.39 to −3.66; p < 0.00001, Fig. 6f) were lower after the LFS intervention than HFS. The inconsistency between the studies using SWS test was moderate (Q = 1.96, I^2^ = 49%) and the inconsistency was low across studies using FOG questionnaires (Q = 0.08, I^2^ = 0%).

### Akinesia

Four studies including 49 participants showed that the akinesia subscore was significantly lower after LFS compared to HFS (mean effect, −1.68; 95% CI, −2.78 to −0.57; p = 0.003) (Fig. 5a). The inconsistency among studies was moderate (Q = 7.19, I^2^ = 72%). However, no significance was observed within medication-on or -off condition separately, except a trend of greater effect in LFS towards significance (Fig. 5b,c).

### Rigidity

Four studies including 49 patients measured the UPDRS-III Item 22 rigidity subscore. No significant difference between the HFS and LFS was observed regardless of the medication condition (p > 0.05) (Fig. 5d–f). High inconsistency was found between the studies.

## Discussion

In this meta-analysis, we provide first-of-its kind evidence of frequency-dependent effects of STN-DBS on different motor symptoms. By analyzing the acute effects of LFS and HFS in STN-DBS on the motor symptoms of patients with PD, our results demonstrated that HFS alleviates tremors better than LFS in the medication-off state, but not in the medication-on state, which is probably due to ceiling-effects on the improvement of tremor in the medication-on state. LFS had greater alleviating effects on akinesia and FOG, and improvement in gait speed compared to HFS. These findings indicates that frequency is an essential parameter for the therapeutic effects of STN-DBS on motor symptoms in PD, and the determination of the frequency setting is critical for the use of DBS in clinical practice.

Pathological oscillations at different power bands (e.g., alpha- (8–12 Hz), beta- (12–30 Hz)/gamma-(30–100 Hz) bands) may be the neural substrate of the frequency-dependent response of DBS^2–4^, contributing to the varied effects between HFS and LFS. For instance, Blumenfeld *et al*. recorded intra-operative local field potentials (LFPs) in the STN, and found that HFS decreased the baseline STN alpha- and beta-band oscillations compared to LFS with equivalent power^4^. Further, 60-Hz DBS amplified alpha/low-beta power (11–15 Hz) and attenuated high-beta power (19–27 Hz), whereas 140 Hz DBS broadly attenuated beta power (15–30 Hz)^2^. Such effects of LFS and HFS on beta-band oscillation attenuation may result in various extent of improvement in akinesia as observed in our analysis.

Similarly, greater reduction in tremors induced by HFS in the medication-off state may arise from the frequency-specific interference effect of STN-DBS on pathological oscillations, that is, HFS attenuates low-beta power, while LFS cannot. Blumenfeld *et al*. proposed that the superior effect of HFS on tremors might be due to reduced coupling in the cortico-striato-STN circuitry with low-beta bands^2^. In addition, other pathological oscillations may be involved in the regulation of tremors. Anzak *et al*. found that low gamma oscillations in the STN are associated with tremor severity in PD. In another study, Beudel *et al*. reported that suppression of the gamma-band in the STN by STN-DBS is inversely related to tremor amplitude. However, as the influence of LFS on gamma-band oscillation is unclear, the frequency-specific effects within gamma power band requires further exploration.

The underlying mechanism through which LFS and HFS influence gait and FOG events may both relate to the pedunculopontine nucleus (PPN), which is located in the caudal pontomesencephalic tegmentum and five mm away from the STN^21,22^, projecting to the cortex and the spinal cord^23^. LFS in the STN may thus affect neural activity in the PPN^24^ with diffused current delivered by the implanted electrode or reciprocal connections between the STN and the PPN. In animal studies, Sitti *et al*. explored the effects of STN-DBS on PPN neural activity and observed that compared to stimulation with 10 Hz or 130 Hz, only 60 Hz STN-DBS produced entrainment of the neural firing pattern in the PPN^25^. Moreover, several studies have comfimed that stimulation of PPN with lower frequency such as 10–25 Hz, sometimes even 60 Hz, had better efficacy of improving gait and locomotion^26–30^. Therefore, LFS targeting the STN may yield a potential propagation to the PPN and induces the changes in the neural activities in PPN, which is needed to be confirmed in future studies by comparing the effects of stimulation targeting STN and PPN.

## Limitations

Two out of four studies on the effects on FOG were excluded. Specifically, in study reported by Xie *et al*.^11^, after the LFS, people perform no any FOG symptom, and thus the scores of FOG were zero, which cannot be used in the meta-analysis^11^. But the findings of Xie *et al*.^11^ was consistent with the results of our analysis, that is, LFS had better efficacy on reducing numbers of FOG^11^. In the other study, no participants presented FOG though the FOG score was measured^17^. The number and the sample sizes of the literatures are thus relatively small and the study designs are of high heterogeneity. Future randomized and double-blinded studies with larger sample sizes are needed to explore explicitly the effects of different frequency bands of on the pathological motor symptoms in population with PD.

## Conclusion

In conclusion, our meta-analysis reveal that HFS of STN-DBS is an effective strategy to alleviate tremors within medication-off condition, and LFS is helpful for severe akinesia and gait disturbances in patients with PD. Although these results are needed to be further confirmed in future, the observed frequency-specific effects can ultimately inform the frequency programming of STN-DBS in the clinic use.
